# Assessing the potential for Crude Oil degradation by Biosurfactant-producing Bacteria isolated from Marine Ecosystems in Nigeria

**DOI:** 10.1099/acmi.0.000953.v6

**Published:** 2026-01-09

**Authors:** O. R. Aina, A. E. Omotayo, G. Efthimiou, O. N. Olaleye, C. E. Oshoma

**Affiliations:** 1 Department of Microbiology, Faculty of Life Sciences, University of Benin, Benin City, Nigeria; 2 Department of Biological Sciences, College of Basic Sciences, Lagos State University of Science and Technology, Ikorodu, Nigeria; 3 Department of Microbiology, Faculty of Science, University of Lagos, Akoka, Nigeria; 4 Centre for Biomedicine, Hull York Medical School, Hull, UK

**Keywords:** biodegradation, biosurfactant, marine ecosystem, optimization

## Abstract

Optimization of petroleum hydrocarbon degradation process in contaminated environments could be feasible using biosurfactant-producing bacteria. The aim of this study was to investigate crude oil degradation potential of biosurfactant-producing bacteria isolated from a marine ecosystem in Nigeria. Sediment and water samples were collected from ten marine locations in Nigeria, and physicochemical analyses were carried out on them. Isolates were identified and screened for biosurfactant production and crude oil degradation after 7 days of incubation. The screened isolates were assayed for biosurfactant production and crude oil degradation for 35 days and analysed every 7 days for changes in pH, OD and total petroleum hydrocarbon content. The strains with the highest yields were identified using PCR-based molecular method. Twenty bacterial species were isolated from the marine locations, and 15 of these isolates showed good potential for biosurfactant production and crude oil degradation. The isolates with the highest biosurfactant production using oil spread and emulsification index tests are *Pseudomonas aeruginosa* Sihong_820_11, *P. aeruginosa* Strain P73 and *Atlantibacter hermannii* Strain K167. In addition, these bacterial isolates have the highest crude oil degradation efficiencies of 87%, 68% and 68%, respectively. The findings revealed that biosurfactant-producing bacteria isolated from marine ecosystems within Nigeria could effectively degrade crude oil in contaminated sites. In addition, bacteria with higher potential for biosurfactant production are more efficient in crude oil degradation.

**Impact Statement**

The study has revealed that Lagos lagoons, beaches and Ilaje creeks contain some indigenous bacterial genera which include *Pseudomonas* sp., *Micrococcus* sp*.*, *Alcaligenes* sp. and *Escherichia* sp. These bacteria can effectively produce biosurfactants and degrade crude oil. A significant knowledge gap is filled by identifying *Atlantibacter hermannii* strain K167, which produces biosurfactants and degrades petroleum hydrocarbons including polyaromatic hydrocarbons up to 65% within 2 weeks of incubation. The findings revealed that biosurfactant production and crude oil degradation efficiencies using *Pseudomonas aeruginosa* strain Sihong_820_11 could reach up to 68% and 87%, respectively, within 35 days of incubation. Additionally, it is established that biosurfactants are involved in environmental, industrial, agricultural and medical applications. They function as food ingredients, additives, emulsifiers, solubilizers and foaming and wetting and antimicrobial agents, preventing food spoilage and growth of foodborne pathogenic bacteria, among others. The results of this study underscore biosurfactant potential environmental benefits. This research not only enhances our understanding of biosurfactant production by different bacterial species but also presents its positive effect on crude oil degradation. Finally, this research presents practical contributions to the achievement of the Sustainable Development Goals 14 and 15 of the United Nations. These goals articulate targets for the conservation and sustainable use of oceans, seas and marine resources, achieving a state whereby the quality and quantity of land resources remain stable within such ecosystems.

## Data Summary

16S rRNA sequences were submitted in GenBank: MAK-S (accession code PQ857166), MID-W (accession code PQ857167) and OKO-S (accession code PQ857168).

## Introduction

Global development has been consistent over the past 10 years because of industrial, infrastructural and technological growth. One of the challenges brought by these advancements is environmental contamination [1,2]. Due to the ongoing contamination from organic, inorganic and xenobiotic substances, ecosystems are now being threatened. One of the main causes of the harm that ecosystems are experiencing is the use of contemporary methods for oil exploration in the crude oil industry. Additionally, the disposal of undesirable materials obtained from the production of crude oil is a worldwide problem since they pollute and disturb terrestrial, aquatic, mangrove and air ecosystems [3,4]. It is crucial to clean up such contaminated areas because of the toxicity, lingering and detrimental effects of these contaminants on living organisms.

Divergent techniques are utilized to eradicate or reduce crude oil pollution, but these techniques are costly, energy-demanding and time-consuming [5]. There was, therefore, a serious search for cheap, simple, fast and eco-friendly techniques. Researchers have proven that micro-organisms through the process called biodegradation can degrade inorganic contaminants such as crude oil [3].

Micro-organisms can be assisted in breaking down these harmful chemicals. Biosurfactant makes the hydrocarbons in crude oil easily available for breakdown; therefore, bacteria that can produce such biosurfactants are better degraders of hydrocarbons [6,8]. Microbial strain enhancement and media supplementation may be some ways to optimize a micro-organism’s production of biosurfactants [9]. Micro-organisms naturally produce metabolites because of both catabolic and anabolic processes within their cells. Improvement is always required if they are to produce these chemicals in more quantity and quickly [10]. Hyper-secretion procedures must be used in order for any strain of microbes to secrete chemicals on a commercial basis. Recombinant DNA engineering and mutagenetic enhancement of micro-organism strains are examples of hyper-secretion approaches [9,10].

In finding solutions to the challenges of hydrocarbon pollution of the ecosystems because of crude oil exploration and exploitation activities, biosurfactants are very active for the enhancement of biodegradation of crude oil. Biosurfactant-producing bacteria can be discovered and exploited to compose products that will enhance the biodegradation of petroleum hydrocarbons in crude oil-impacted ecosystems, and optimization process could be feasible using mutant strains of biosurfactant-producing bacteria.

A systematic collaborative study is required to investigate and utilize the mutant strains of bacteria that produce biosurfactants for the improvement of crude oil degradation in order to help meet the Sustainable Development Goals 14 and 15 of the UN.

## Methods

### Description of sampling locations

**Fig. 1.**
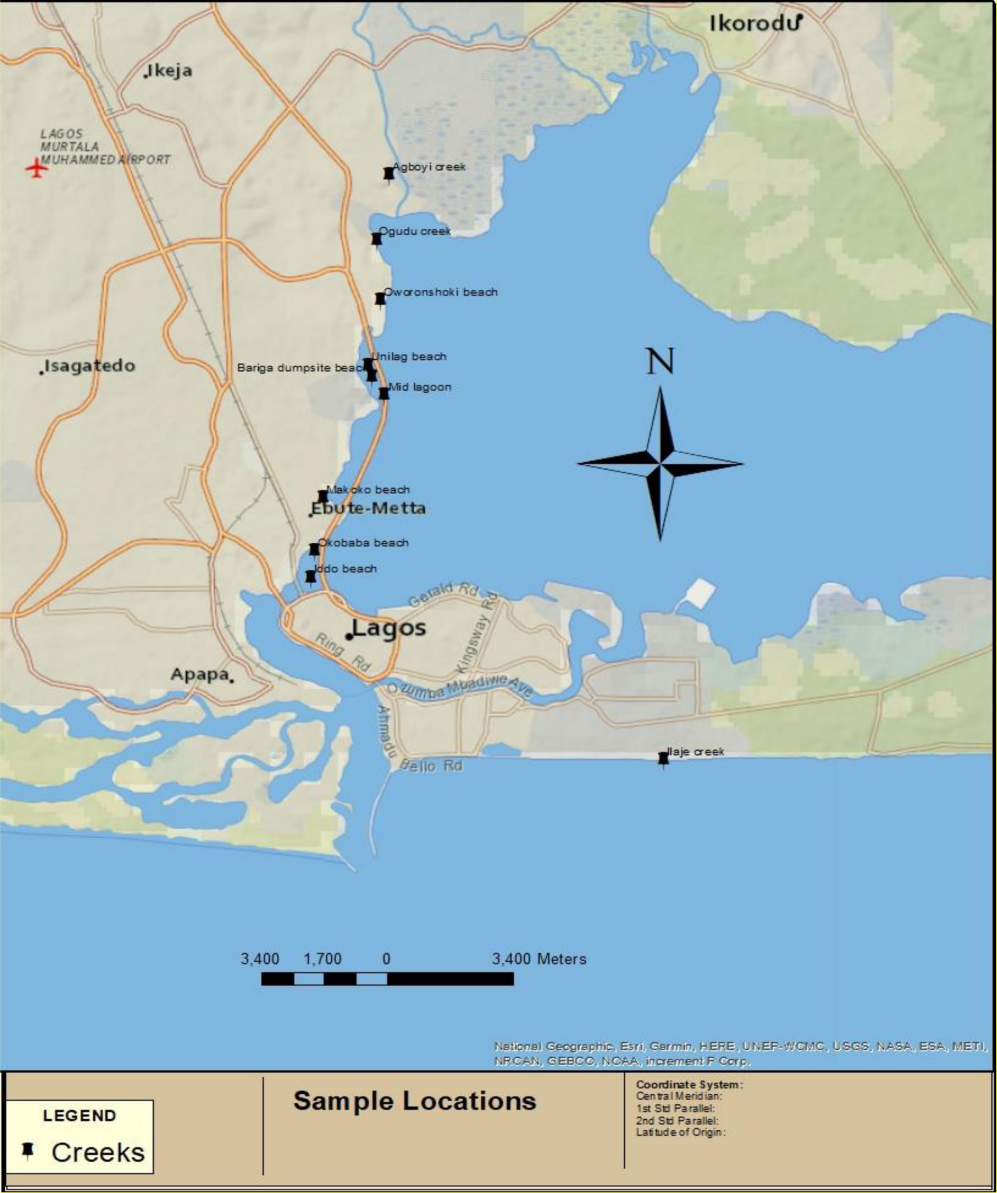
Sketch map of Lagos lagoons showing some of the sampling locations (software reference: ArcGIS 12).

The marine locations used for this study were beaches, creeks and lagoons from Lagos State and Ondo State, Nigeria. These marine locations and their Global Positioning System are shown in Table 1, and a simple sketch map is shown in Fig. 1 for further elaboration [11]. Some of these locations such as Bariga dumpsite beach, Oworonshoki beach and Ilaje creek are confirmed to have been exposed to crude oil contamination.

**Table 1.** Sampling locations and GPS location values

S/no.	Sampling location	GPS value
1	Ogudu creek	6° 33′ 49″ N 3° 24′ 26″ E
2	Mid lagoon	6° 31′ 32″ N 3° 24′ 33″ E
3	Unilag beach	6° 31′ 5″ N 3° 24′ 11″ E
4	Bariga dumpsite beach	6° 31′ 32″ N 3° 24′ 2″ E
5	Oworonshoki beach	6° 32′ 52″ N 3° 24′ 29″ E
6	Agboyi creek	6° 34′ 5″ N 3° 24′ 39″ E
7	Makoko beach	6° 29′ 39″ N 3° 23′ 45″ E
8	Okobaba beach	6° 28′ 57″ N 3° 23′ 33″ E
9	Iddo beach	6° 28′ 11″ N 3° 23′ 3″ E
10	Ilaje creek	6° 25′ 24″ N 4° 75′ 23″ E

### Collection of water and sediment samples

Between August 2020 and August 2021, ten water and ten sediment samples were taken from ten marine locations in Lagos State and Ondo State, one water sample and one sediment sample per location, at various intervals (months). The water samples were collected into sterile containers using an A-frame derrick equipped with a multipurpose winch and cable system. Using a Van Veen grab sampler, sediment samples were carefully taken and placed in sterile Ziplock containers. These were immediately stored in the laboratory’s refrigerator at 4 °C [12,13]. The water samples taken at the sites are listed in Table 1 and represented as Ogudu W, Mid lagoon W, Unilag beach W, DS Bariga W, Oworo W, Agboyi W, Makoko W, Okobaba W, Iddo W and Ilaje W. The sediment samples were represented as Ogudu S, Mid lagoon S, Unilag beach S, DS Bariga S, Oworo S, Agboyi S, Makoko S, Okobaba S, Iddo S and Ilaje S.

### Sample preparation

Five grams (5.0 g) each of the sediment samples was suspended in 5.0 ml of distilled water and stirred for 30 min, and the aliquot was used for the physicochemical analysis, while for water samples, physicochemical analysis was done directly according to the standard American Public Health Association (APHA) (1995), 19th edition.

### Physicochemical analysis of water and sediment samples

The physicochemical parameters analysed included turbidity, temperature, pH, total organic matter, total alkalinity, total acidity, chloride, phosphate, chemical oxygen demand, biological oxygen demand (BOD), dissolved oxygen, oil and grease, total dissolved solids and heavy metals, among others.

### Enrichment of crude oil-degrading and biosurfactant-producing bacteria 

Ten grams each of the sediment samples and 10.0 ml each of the water samples were suspended in 90.0 ml of distilled water making the stock solution; afterwards, 10.0 ml of the stock solutions was transferred into 90.0 ml of mineral salt medium (MSM) containing 1.0 g of (NH_4_)2SO_4_, 0.8 g of K_2_HPO_4_, 0.2 g of KH2PO_4_, 0.2 g of Mg SO_4_.7H_2_O, 0.1 g of CaCl_2_.2H_2_O, 0.005 g of Fe SO_4_.7H_2_O, 1.0 g of NA_2_HPO_4_.7H2O, 0.05 g of NaCl, 1 ml of trace elements and 1 ml of sterile crude oil as the sole carbon and energy source in 1,000 ml of distilled water at pH 7.0±0.2. The negative control sample contained 1 ml crude oil and 100 ml sterile MSM. The cultures and the control were incubated in an orbital shaker at 150 r.p.m. at 30 °C. Samples were subjected to five transfers of enrichment culture steps to attain and enhance a well-adapted crude oil-degrading enriched bacteria and also to screen out the non-degraders. Each transfer took place every 2 weeks [14,15].

### Isolation of crude oil-degrading and biosurfactant-producing bacteria and morphological characterization

A 0.1 ml of each sample dilution of the last (fifth) transfer of the enrichment cultures was spread on nutrient agar, and the plates were incubated at 37 °C for 24 h. Each inoculated plate was assessed for bacterial growth, taking into account colony morphology such as opacity, margins, elevation, shape and cell arrangement using the naked eye and microscope. The discrete colonies were further streaked on fresh nutrient agar and incubated at 37 °C for 24 h to obtain pure cultures. Gram staining was performed to identify bacteria based on their cell wall composition [16].

### Biochemical characterization of the bacterial isolates

Biochemical characterization of 20 isolated bacterial strains was performed through the following tests: glucose, lactose, H_2_S production, production of gas, motility, indole, urease, citrate, oxidase, catalase, coagulase, mannitol, spore forming and Voges–Proskauer [17].

### Screening of bacterial isolates for biosurfactant production

A 1-day-old broth culture of the purified pure isolates was centrifuged at 10,000 r.p.m. for 10 min, and the supernatant (cell-free cultures) was subjected to a preliminary screening for biosurfactant production using oil spreading test, drop collapse test and emulsification index test [18].

### Oil spread test

Forty millilitres (40 ml) of sterile distilled H_2_O was placed into a sterile Petri plate, and 20 µl sterile crude oil was introduced. The crude oil was allowed to spread throughout the surface, and afterwards, 10 µl of the culture supernatant was gently introduced. The blank consisted of sterile distilled H_2_O and crude oil. If a biosurfactant is present in the culture, the oil is displaced and a clearing zone is formed. The absence of a clearing zone is an indication of a negative reaction [19].

### Drop collapse test 

A 20 µl supernatant of each sample was mixed with 5 µl methylene blue, and then one drop of the mixture was placed on a crude oil surface, and the outcome of the drop was observed on the surface after 60 s. The collapse of the drop indicated the presence of biosurfactant in the culture, and the absence of a collapse is an indication of a negative reaction. Methylene blue was added solely to serve as a control [19].

### Emulsification index (E_24_) test

A 6 ml of sterile crude was mixed with 4 ml culture supernatant in a test tube, and the mixture was vortexed at high speed for 2 min and was allowed to stand for 24 h [17]. The percentage of emulsification index was calculated as follows:


$$E_{24}\;Index\%=\frac{Height\;of\;emulsion\;formed}{Total\;height\;of\;solution}\times 100.$$

### Screening for crude oil degradation of the pure isolates using 2,6-dichlorophenolindophenol redox dye

Isolates that tested positive for biosurfactant production were further screened for crude oil degradation using 2,6-dichlorophenolindophenol (DCPIP) test. The principle DCPIP redox indicator is that the more reduction reactions, the more the colour change from blue to colourless. One millilitre of isolate supernatant from the culture broth was introduced into 100 ml of MSM containing 0.5 ml of crude oil and 0.2 ml of DCPIP redox dye in 250 ml conical flasks. The culture suspension was incubated at 28 °C for 7 days on an orbital shaker at a speed of 180 r.p.m. One of the flasks was uninoculated and served as a control; flasks were monitored for colour change (from deep blue to colourless) on days 0, 3, 5 and 7 [20,21].

### Screening of bacterial isolates for crude oil degradation using OD

Fifteen isolates that tested positive for biosurfactant production in the three tests were screened for crude oil degradation by investigating the bacterial growth rate which was determined using the OD/medium absorbance. A 1 ml isolate suspension contains 100 ml of MSM, 1 ml of crude oil and 1 ml of inocula in 250 ml conical flask, and the control was aseptically dispensed into the cuvette of UV-VIS spectrophotometer; the OD was measured using UV-VIS spectrophotometer at 600 nm on days 0, 3, 5 and 7 [21,22].

### Assay for crude oil degradation and biosurfactant production potentials

Seven isolates with high potential for biosurfactant production and crude oil degradation were further investigated to select the three most efficient biosurfactant producers and crude oil degraders using the following parameters: bacterial growth (OD), changes in pH, biosurfactant concentration and crude oil content [total petroleum hydrocarbon (TPH)]. One hundred millilitres of MSM was aseptically prepared in 250 ml conical flasks, 1 ml of sterile crude oil was added and 1 ml of inocula of the organisms was introduced into the flasks; one of the flasks was uninoculated and served as control. The samples were incubated on a rotary shaker at 30 °C and 180 r.p.m. for 35 days. These were analysed weekly for 35 days. The parameters were analysed every 7-day interval for 35 days, and readings were taken in triplicate and the mean values were recorded [22].

### Assay for crude oil degradation potentials using changes in OD

The growth of the seven (efficient) isolates was determined using the OD/medium absorbance during crude oil degradation for 35 days; 1 ml of each isolate suspension and the control was aseptically dispensed into the cuvette of UV-VIS spectrophotometer, and the OD was measured using UV-VIS spectrophotometer at 600 nm on days 0, 7, 14, 21, 28 and 35 [22].

### Assay for crude oil degradation potentials using changes in the pH of the cultures

Ten millilitres of each isolate suspension was aseptically dispensed into fresh and sterile 100 ml conical flask, and the pH was measured by inserting a digital pH meter-Walfront Smart Sensor As 218 made by JXCT, China, into the medium and the readings were taken every 7-day interval for 35 days [22].

### Assay for crude oil degradation potentials using changes in TPH content

Ten millilitres of each isolate suspension was aseptically dispensed into sterile 250 ml beaker, and 35 ml of n-hexane and 15 ml of dichloromethane were added. The mixture was transferred into a 500 ml separating funnel and was shaken vigorously for 2 min and was allowed to settle and separate for 5 min. The solvent layer was drained after 5 min into a fresh and cleaned beaker, and the solvent was later dispensed into a column containing silica gel and magnesium sulphate which were used as the solid phase. The extracted solvent was stored in sterile vials for quantification using GC-MS. The steps were repeated weekly for 35 days [22].

### Assay for biosurfactant production potentials using oil spread and emulsification index (E_24_) tests

One millilitre of each isolate suspension was transferred aseptically into a test tube and was centrifuged at 10,000 r.p.m. for 10 min; the supernatant was assayed for biosurfactant production abilities using oil spread test (OST) and emulsification index (E_24_) test. These were analysed weekly for 35 days [23].

### Assay for biosurfactant production using OST

Sterile distilled water (40 ml) was placed in a Petri dish of 15 cm diameter, 20 µl of crude oil was gently added to spread on the surface and then 10 µl of the culture supernatant was gently introduced. Immediate appearance of a clear zone confirmed the presence of biosurfactant in the culture supernatant, and the diameter of the clear zone was measured and recorded. Sterile distilled water was used as the control, and the steps were repeated at 7-day interval for 35 days [23].

### Assay for biosurfactant production using emulsification index (E_24_) test

Crude oil (6 ml) was mixed with 4 ml culture suspension in a test tube, and the mixture was vortexed at high speed for 2 min and was allowed to stand for 24 h. The percentage of emulsification index was calculated as follows:


$$E_{24}\;index\%=\frac{Height\;of\;emulsion\;formed}{Total\;height\;of\;solution}\times 100.$$

These steps were repeated at every 7-day interval for 35 days [23].

### Molecular characterization of bacterial isolates

For the molecular characterization of samples, the isolates MAK-S, MID-W and OKO-S were selected based on their high potentials for biosurfactant production and crude oil degradation. These three bacterial isolates were inoculated on nutrient agar and incubated for 24 h at 37 °C to obtain pure isolates.

### DNA extraction

The genomic DNA of each of the three isolates was extracted using cetyltrimethylammonium bromide [24] with slight modifications to optimize the quality of DNA.

### PCR amplification

The extracted DNA was used as a template for 16S rRNA PCR (Bio-Rad T100 PCR Thermal) using 271F (5′-AGAGTTTGATCCTGGCT CAG-3′) and 1492R (5′-GGTTACCTTGTTACGACTT-3′) for MAK-S and PA-SS-F (5-′GGGGGATCTTCGGACCTCA-3′) and PA-SS-R (5′- TCCTTAGAGTGCCCACCCG-3′) for MID-W and OKO-S [24].

### Phylogenetic analysis

For the assembly of 16S rRNA sequences, Geneious version 9.0.5 was used. The sequences were identified through the blast tool on the National Centre for Biotechnology Information (NCBI). Quality trimming of the sequenced data was performed using FinchTV software, and multiple sequence alignment was carried out using the ClustalW algorithm. The phylogenetic tree was inferred using the maximum likelihood method with 1,000 bootstrap replication value, using MEGAII software. All three sequences, MAK-S, MID-W and OKO-S, were submitted in the NCBI GenBank.

### Data analysis of the results

The experiments were done in three replicates and three readings were recorded for each experiment. Means and sd of the data were calculated. Means of the physicochemical and other bacteriological parameters were compared by using descriptive analysis.

## Results

### Physicochemical properties of water and sediment samples

Ten water samples with varying characteristics have been recorded in Table 2. The appearance of all the water samples was clear except for the sample from Agboyi, which was slightly dirty. The highest turbidity of 26.21 nephelometric turbidity unit (NTU) was recorded in a sample from Okobaba which confirms the high heterotrophic count recorded in this sample, while the least turbidity of 13.62 NTU was recorded in a sample from Oworo.

**Table 2.** Physicochemical properties of marine water samples

Parameter	A	M	Ow	Og	DSB	Id	Il	Ok	U	ML
Appearance	SD	C	C	C	C	C	C	C	C	C
Form	L	L	L	L	L	L	L	L	L	L
Colour	CL	CL	CL	CL	CL	CL	CL	CL	CL	CL
Turbidity	17.65±0.10	15.38±0.04	13.62±0.04	14.36±0.13	15.21±0.12	15.34±0.11	16.84±0.10	26.21±0.09	18.41±0.08	16.92±0.07
Temperature (°C)	29.2±0.09	28.5±0.00	29.3±0.00	28.3±0.02	29.2±0.08	28.9±0.07	29.2±0.06	29.6±0.05	28.9±0.04	28.5±0.03
pH	7.34±0.70	7.50±0.10	7.76±0.20	7.45±0.35	7.54±0.03	7.86±0.40	7.74±0.50	7.34±0.60	8.14±0.70	8.34±0.30
EC (µScm^1^)	11.22±0.10	12.46±0.01	22.96±0.03	34.3±0.03	43.8±0.02	44.3±0.09	39.8±0.08	38.55±0.07	16.24±0.06	17.61±0.05
Total organic carbon (%)	4.95±0.50	3.21±0.00	20.92±0.05	10.44±0.05	6.99±0.50	13.73±0.04	11.46±0.03	7.48±0.02	15.63±0.01	18.43±0.50
Total organic matter (%)	8.56±0.01	5.55±0.02	36.17±0.00	18.05±0.02	12.09±0.10	23.74±0.20	19.81±0.30	12.93±0.40	27.02±0.50	31.86±0.60
Total alkalinity (mg l^−1^)	42.30±1.00	62.42±2.00	35.61±3.05	49.31±4.01	36.81±1.70	35.34±2.30	34.88±3.40	44.35±4.50	68.5±3.50	73.56±2.50
Total acidity (mg l^−1^)	18.66±0.20	14.57±0.02	12.53±0.04	10.36±0.00	9.76±0.03	17.61±0.10	10.41±0.20	11.46±0.09	19.42±0.75	20.52±0.10
Chloride (mg l^−1^)	168.45±8.20	176.5±5.01	153.4±5.01	145.31±7.40	159.76±5.30	139.42±6.20	152.43±5.01	163.16±7.40	175.31±5.30	176.48±6.40
Salinity (mg l^−1^)	24.34±3.50	29.24±2.60	18.76±2.00	24.63±2.03	27.82±2.70	31.42±2.85	29.86±3.00	24.92±2.00	36.92±2.03	16.43±2.70
Nitrate (mg l^−1^)	5.24±0.50	4.93±0.02	3.41±0.00.01	2.96±0.33	4.75±0.20	5.62±0.21	3.96±0.22	3.58±0.23	4.63±0.24	2.63±0.25
Ammoniacal nitrogen (mg l^−1^)	1.64±0.00	2.51±0.01	1.93±0.01	1.04±0.01	0.94±0.10	1.14±0.10	1.27±0.01	1.38±0.00	1.04±0.01	0.94±0.02
Total nitrogen (mg l^−1^)	0.081±0.02	0.033±0.00	0.041±0.01	0.022±0.00	0.024±0.00	0.032±0.00	0.026±0.01	0.014±0.01	0.013±0.01	0.015±0.00
Sulphate (mg l^−1^)	74.6±0.20	94.81±0.10	63.41±0.03	82.46±0.57	78.44±0.54	79.84±0.05	81.39±0.06	75.64±0.08	69.39±0.20	82.51±0.10
Phosphate (mg l^−1^)	2.01±0.20	0.98±0.01	1.25±0.03	1.36±0.01	1.72±0.03	1.68±0.03	1.84±0.02	1.94±0.01	1.49±0.00	1.33±0.02
COD (mg l^−1^)	42.6±4.00	54.92±7.40	36.71±6.04	63.4±7.06	58.3±5.50	70.41±6.50	46.74±7.50	61.82±7.05	72.31±5.00	69.45±6.03
BOD (mg l^−^)	12.68±2.00	29.45±4.50	9.89±3.03	13.74±4.10	14.81±3.20	18.32±3.50	10.63±2.50	12.41±2.03	14.82±3.10	13.54±3.05
Dissolved oxygen (mg l^−1^)	6.87±0.52	4.95±0.51	3.63±0.06	4.46±0.03	4.79±0.05	3.67±0.60	3.98±0.50	5.93±0.51	8.47±0.52	10.3±0.05
Oil and grease (mg l^−1^)	1.63±0.02	4.22±0.60	2.04±0.01	1.07±0.01	1.28±0.10	1.42±0.01	1.18±0.02	2.14±0.03	1.43±0.00	2.56±0.04
TD S (mg l^−1^)	134.7±1.50	156.1±7.20	196.3±1.40	236.9±0.07	1384.7±1.10	672.6±1.50	1293.7±1.50	215.9±1.00	1521.3±1.50	2014.5±1.00
Sodium (mg l^−1^)	4.35±0.50	5.83±0.50	3.89±0.30	2.47±0.03	4.46±0.50	5.14±0.60	3.89±0.50	2.68±0.40	13.45±0.30	15.75±0.20
Lead (mg l^−1^)	0.361±0.01	0.042±0.00	0.121±0.07	0.83±0.00	0.276±0.01	0.096±0.00	0.241±0.01	0.307±0.10	2.56±0.11	1.063±0.10
Potassium (mg l^−1^)	2.64±0.10	4.34±0.02	1.68±0.07	3.04±0.03	26.3±0.50	29.6±0.06	22.3±0.07	12.26±0.50	0.002±0.00	1.24±0.01
Zinc (mg l^−1^)	0.259±0.00	0.364±0.01	0.481±0.03	0.524±0.01	0.674±0.01	0.425±0.03	0.826±0.02	133.8±3.01	96.35±1.35	83.62±1.75
Cadmium (mg l^−1^)	0.074±0.02	0.092±0.00	0.044±0.03	0.063±0.00	0.083±0.00	0.078±0.01	0.006±0.00	0.46±0.10	0.65±0.15	0.38±0.01
Arsenic (mg l^−1^)	0.003±0.00	0.005±0.00	0.002±0.03	0.004±0.00	0.016±0.00	0.027±0.01	0.003±0.00	0.014±0.01	0.005±0.01	0.002±0.00
Chromium (mg l^−1^)	0.061±0.01	0.084±0.01	0.028±0.10	0.018±0.01	0.038±0.00	0.058±0.00	0.074±0.01	1.121±0.10	1.036±0.12	0.941±0.01
Iron (mg kg^−1^)	0.281±0.02	0.406±0.02	0.904±0.01	0.524±0.01	0.574±0.01	0.731±0.01	0.824±0.02	0.128±0.01	1.176±0.01	1.006±0.10
Magnesium (mg kg^−1^)	7.89±0.51	10.24±0.68	6.29±0.01	8.26±0.05	12.43±0.01	8.49±0.52	5.99±0.09	10.94±0.78	4.21±0.22	3.94±0.02

A, Agboyi; C, clear; CL, colourless; DSB, DS Bariga; Id, Iddo; Il, Ilaje; L, liquid; M, Makoko; ML, midlagoon; Og, Ogudu; Ok, Okobaba; Ow, Oworo; SD, slightly dirty; U, Unilag.

Ten sediment samples with varying characteristics from ten marine locations have been recorded in Table 3. The highest pH of 7.90 was recorded in a sample from Midlagoon, while the lowest pH of 7.26 was recorded in a sample from Oworo; the highest value of oil and grease of 6.14 mg kg^−1^ was recorded in a sample from Okobaba, while the lowest value of oil and grease of 1.24 mg kg^−1^ was recorded in a sample from Midlagoon; the highest value of salinity of 18.3 mg kg^−1^ was recorded in a sample from Midlagoon, while the lowest value of salinity of 3.44 mg kg^−1^ was recorded in samples from Makoko and Unilag beach; the highest value of BOD of 74.43 mg kg^−1^ was recorded in a sample from Okobaba, while the lowest value of BOD of 3.84 mg kg^−1^ was recorded in a sample from Iddo.

**Table 3.** Physicochemical properties of marine sediment samples

Parameter	A	M	Ow	Og	DSB	Id	Il	Ok	U	ML
Appearance	Wet/black	Wet/black	Wet/black	Wet/black	Wet/black	Wet/black	Wet/black	Wet/black	Wet/black	Wet/black
Form	Semi-solid	Semi-solid	Semi-solid	Semi-solid	Semi-solid	Semi-solid	Semi-solid	Semisolid	Semi-solid	Semi-solid
Colour	Black	Black	Black	Black	Black	Black	Black	Black	Black	Black
Temperature (°C)	26.9±3.10	27.4±3.20	28.5±3.30	27.8±3.40	28.9±3.50	26.3±3.40	27.4±3.30	28.1±3.20	27.8±3.10	26.1±3.00
pH	7.44±0.51	7.35±0.52	7.26±0.53	7.29±0.54	7.42±0.55	7.40±0.56	7.38±0.57	7.30±0.58	7.65±0.59	7.90±0.60
Electrical conductivity (µScm­¹)	3.56±0.10	2.08±0.11	7.04±0.12	4.41±0.13	3.98±0.14	0.58±0.15	3.08±0.16	5.84±0.17	34.2±0.18	20.4±0.19
Total organic carbon (%)	5.37±0.50	4.33±0.50	8.45±0.51	6.31±0.53	5.87±0.52	11.3±0.53	2.76±0.50	7.56±0.51	6.34±0.52	6.62±0.53
Total organic matter (%)	9.28±1.00	7.49±1.03	14.61±1.05	10.91±1.01	7.51±1.02	19.54±1.04	4.77±0.98	13.07±0.78	10.94±1.00	9.72±0.83
Total alkalinity (mg kg^−1^)	72.6±7.20	68.9±7.02	84.6±6.94	79.4±7.40	96.1±7.14	76.9±6.95	87.4±7.02	63.5±7.06	86.5±7.12	99.31±7.06
Total acidity (mg kg^−1^)	25.7±3.01	21.8±3.03	9.6±3.05	20.56±3.02	18.81±3.04	16.85±3.00	10.91±3.01	18.41±3.02	37.6±3.50	25.46±3.40
Chloride (mg kg^−1^)	112±0.60	96±0.50	184±0.71	136±0.93	126±0.53	175±0.55	144±0.82	159±0.78	147±0.65	184±0.50
Salinity (mg kg^−1^)	4.94±0.01	3.44±0.01	5.92±0.02	4.54±0.15	5.29±0.13	3.75±0.07	3.93±0.07	16.44±0.17	3.44±0.01	18.3±0.19
Nitrate (mg kg^−1^)	1.03±0.01	0.99±0.00	2.14±0.03	2.89±0.05	1.24±0.01	2.49±0.01	2.56±0.02	0.89±0.04	17.52±0.07	19.36±0.08
Ammoniacal nitrogen (mg kg^−1^)	9.38±0.06	7.61±0.05	6.34±0.08	8.84±0.07	5.86±0.04	6.21±0.05	4.93±0.03	2.26±0.01	11.56±0.06	9.85±0.07
Total nitrogen (%)	0.32±0.01	0.18±0.09	0.29±0.02	0.16±0.08	0.22±0.03	0.14±0.07	0.16±0.04	0.26±0.06	0.29±0.05	0.12±0.01
Sulphate (mg kg^−1^)	14.2±0.29	12.42±0.25	216±0.26	187±0.28	167±0.24	175±0.26	146±0.27	156±0.23	156±0.25	125±0.26
Phosphate (mg kg^−1^)	0.52±0.00	68.4±0.07	4.99±0.01	3.88±0.00	4.56±0.02	5.16±0.01	5.50±0.01	0.56±0.02	0.42±0.01	0.63±0.01
COD (mg kg^−1^)	26.23±0.04	38.1±0.03	29.96±0.02	34.5±0.05	56.11±0.06	24.7±0.03	50.2±0.03	161.47±0.17	17.83±0.02	34.2±0.05
BOD (mg kg^−1^)	10.46±0.06	8.42±0.05	5.68±0.04	6.93±0.07	12.24±0.08	3.84±0.01	23.1±0.09	74.43±1.02	5.89±0.02	6.43±0.02
Dissolved oxygen (mg kg^−1^)	3.37±0.04	2.81±0.00	2.41±0.01	3.41±0.02	2.74±0.03	1.76±0.04	4.61±0.03	5.16±0.02	4.26±0.01	5.32±0.04
Oil and grease (mg kg^−1^)	1.25±0.00	1.75±0.00	1.44±0.00	1.39±0.00	1.58±0.00	2.9±0.01	4.22±0.01	6.14±0.10	2.74±0.01	1.24±0.00
Iron (mg kg^−1^)	0.022±0.01	0.042±0.00	0.09±0.01	0.14±0.00	0.08±0.00	0.03±0.00	0.06±0.00	0.08±0.00	0.04±0.00	0.26±0.01
Sodium (mg kg^−1^)	2.32±0.03	6.74±0.05	5.21±0.04	3.94±0.03	2.94±0.02	4.87±0.04	3.94±0.03	2.64±0.02	1.52±0.01	6.96±0.06
Lead (mg kg^−1^)	0.062±0.02	0.042±0.00	0.09±0.02	0.14±0.00	0.08±0.00	0.03±0.00	0.06±0.01	0.08±0.01	0.04±0.00	0.26±0.01
Potassium (mg kg^−1^)	22.56±0.05	14.61±0.03	19.71±0.05	34.1±0.50	26.3±0.04	29.6±0.50	22.3±0.05	12.26±0.01	0.002±0.00	1.24±0.01
Zinc (mg kg^−1^)	29.42±0.0.50	4.47±0.05	34.62±0.60	56.2±1.00	38.1±0.70	40.4±0.50	67.1±1.10	0.18±0.01	24.5±0.18	10.6±0.20
Cadmium (mg kg^−1^)	0.15±0.01	0.33±0.02	0.22±0.03	0.17±0.00	0.11±0.01	0.19±0.02	0.27±0.03	0.14±0.00	35.7±0.50	4.52±0.10
Arsenic (mg kg^−1^)	0.03±0.00	0.05±0.00	0.09±0.00	0.04±0.00	0.06±0.00	0.09±0.00	0.04±0.00	0.003±0.00	0.001±0.00	0.002±0.00
Chromium (mg kg^−1^)	15.2±0.50	0.93±0.02	1.86±0.25	0.85±0.01	0.59±0.01	0.79±0.02	0.86±0.02	0.005±0.00	0.001±0.00	5.14±0.25
Copper (mg kg^−1^)	0.046±0.01	0.16±0.10	0.26±0.11	0.42±0.12	0.56±0.11	0.36±0.10	0.93±0.15	0.004±0.00	0.03±0.00	0.02±0.00
Magnesium (mg kg^−1^)	6.24±0.01	6.91±0.00	29.3±0.03	56.4±0.30	31.4±0.03	49.3±0.01	21.56±0.03	24.16±0.03	4.58±0.04	2.36±0.02

A, Agboyi; DSB, DS Bariga; Id, Iddo; Il, Ilaje; M, Makoko; ML, midlagoon; Og, Ogudu; Ok, Okobaba; Ow, Oworo; U, Unilag.

### Macroscopic and microscopic characteristics of isolates

All isolates formed colonies on nutrient agar. A total of 20 pure bacterial isolates (10 from water samples and 10 from sediment samples) were obtained from 10 different locations of marine ecosystem as shown in Table 4. Colony morphology revealed 14 isolates as opaque and 6 as translucent, the elevation was mostly flat, the margin was mostly irregular and they mostly appeared in clusters. The isolates are mostly rod-shaped except for three, which were coccobacilli and one cocci.

**Table 4.** Morphological characteristics of bacterial isolates

S/N	Colony	Structure elevation margin			Shape cell arrangement	
1	MAK-W	Opaque	Flat	Irregular	Rod	Chains
2	OGU-W	Translucent/opaque	Flat	Entire	Rod	Clusters
3	OWO-W	Opaque	Convex	Entire	Cocci	Pairs
4	OWO-S	Translucent/opaque	Flat	Entire	Rod	Clusters
5	MID-W	Opaque	Flat	Irregular	Rod	Singly/clusters
6	MID-S	Translucent/opaque	Convex	Irregular	Rods/coccobacilli	Clusters
7	OGU-S	Opaque	Flat	Irregular	Rod	Singly/clusters
8	ILA-S	Translucent/opaque	Convex	Irregular	Rods/coccobacilli	Clusters
9	ILA-W	Translucent/opaque	Flat	Entire	Rod	Clusters
10	UNI-W	Opaque	Flat	Irregular	Rod	Singly/clusters
11	AGBO-S	Opaque	Flat	Irregular	Rod	Singly/clusters
12	DSB-W	Opaque	Flat	Irregular	Rod	Singly/clusters
13	DSB-S	Opaque	Flat	Irregular	Rod	Singly/clusters
14	IDDO-W	Opaque	Flat	Irregular	Rod	Singly/clusters
15	OKO-W	Opaque	Flat	Irregular	Rod	Singly/clusters
16	OKO-S	Opaque	Flat	Irregular	Rod	Singly/clusters
17	IDDO-S	Opaque	Flat	Irregular	Rod	Singly/clusters
18	AGBO-W	Translucent/opaque	Convex	Irregular	Rods/coccobacilli	Clusters
19	MAK-S	Opaque	Flat	Irregular	Rod	Singly/clusters
20	UNI-S	Opaque	Flat	Irregular	Rod	Singly/clusters

Biochemical characterization in Table 5 revealed that most isolates were positive for motility, catalase, oxidase, citrate, mannitol and pigmentation tests. Also, most isolates tested negative for glucose fermentation, spore formation, lactose and Voges–Proskauer test. Based on Gram staining, 18 out of 20 isolates were Gram-positive, and two were Gram-negative.

**Table 5.** Biochemical characterization of the bacterial isolates from water and sediment samples

Isolates	Glucose	Lactose	H_2_s	Gas	Motility	Indole	Urease	Citrate	Oxidase	Catalase	Coagulase	Mannitol	Spore formers	Pigmentation	Voges–Proskauer	Gram stain	Suspected organism
MAK-W	−	−	−	+	+	−	−	+	+	+	−	+	−	+ Yellow–green	−	GNB	*Pseudomonas aeruginosa*
OGU-W	−	−	−	+	+	−	−	+	+	+	−	+	−	+ Green	−	GNB	*Pseudomonas* sp.
OWO-W	+	+	−	−	−	−	+	−	+	+	−	−	−	+ Yellow	−	GPC	*Micrococcus* sp.
OWO-S	−	−	−	+	+	−	−	+	+	+	−	+	−	+ Green	−	GNB	*Pseudomonas* sp.
MID-W	−	−	−	+	+	−	−	+	+	+	−	+	−	+ Blue–green	−	GNB	*Pseudomonas aeruginosa*
MID-S	−	−	−	−	+	−	−	+	+	+	−	−	−	−	−	GNB	*Alcaligenes faecalis*
OGU-S	−	−	−	+	+	−	−	+	+	+	−	+	−	+ Blue–green	−	GNB	*Pseudomonas aeruginosa*
ILA-S	−	−	−	−	+	−	−	+	+	+	−	−	−	−	−	GNB	*Alcaligenes faecalis*
ILA-W	−	−	−	+	+	−	−	+	+	+	−	+	−	+ Green	−	GNB	*Pseudomonas* sp.
UNI-S	−	−	−	+	+	−	−	+	+	+	−	+	−	+ Yellow–green	−	GNB	*Pseudomonas aeruginosa*
UNI-W	−	−	−	+	+	−	−	+	+	+	−	+	−	+ Yellow–green	−	GNB	*Pseudomonas aeruginosa*
AGBO-S	−	−	−	+	+	−	−	+	+	+	−	+	−	+ Blue–green	−	GNB	*Pseudomonas aeruginosa*
DSB-W	−	−	−	+	+	−	−	+	+	+	−	+	−	+ Yellow–green	−	GNB	*Pseudomonas aeruginosa*
DSB-S	−	−	−	+	+	−	−	+	+	+	−	+	−	+ Blue–green	−	GNB	*Pseudomonas aeruginosa*
IDDO-W	−	−	−	+	+	−	−	+	+	+	−	+	−	+ Blue–green	−	GNB	*Pseudomonas aeruginosa*
OKO-W	−	−	−	+	+	−	−	+	+	+	−	+	−	+ Blue–green	−	GNB	*Pseudomonas aeruginosa*
OKO-S	−	−	−	+	+	−	−	+	+	+	−	+	−	+ Blue–green	−	GNB	*Pseudomonas aeruginosa*
IDDO-S	−	−	−	+	+	−	−	+	+	+	−	+	−	+ Blue–green	−	GNB	*Pseudomonas aeruginosa*
AGBO-W	−	−	−	+	+	−	−	+	+	+	−	+	−	+ Yellow–green	−	GNB	*Pseudomonas aeruginosa*
MAK-S	−	−	−	−	+	−	−	+	+	+	−	−	−	−	−	GNB	*Escherichia* sp.

+, positive reaction; −, negative reaction; AGBO-S, Agboyi sediment; AGBO-W, Agboyi water; DSB-S, Bariga dumpsite beach; DSB-W, Bariga dumpsite beach; GNB, Gram-negative bacilli; GPC, Gram-positive cocci; IDDO-S, Iddo sediment; IDDO-W, Iddo water; ILA-S, Ilaje sediment; ILA-W, Ilaje water; MAK-S, Makoko sediment; MAK-W, Makoko water; MID-S, Midlagoon sediment; MID-W, Midlagoon water; OGU-S, Ogudu sediment; OGU-W, Ogudu water; OKO-S, Okobaba sediment; OKO-W, Okobaba water; OWO-S, Oworonshoki sediment; OWO-W, Oworonshoki water; sp., specie; UNI-S, Unilag beach sediment; UNI-W, Unilag beach water.

### Initial biosurfactant production of the pure isolates

The results of the oil spread and drop collapse tests in Table 6 revealed that 15 isolates with varying clear zone diameters tested positive. These isolates include *Pseudomonas* sp.-ILA-W, *Pseudomonas* sp.-OGU-W, *Pseudomonas aeruginosa*-IDDO-S, *P. aeruginosa*-IDDO-W, *Escherichia* sp.-MAK-S, *P. aeruginosa*-MAK-W, *Pseudomonas* sp.-OWO-S, *Micrococcus* sp.-OWO-W, *P. aeruginosa*-UNI-W, *P. aeruginosa*-AGB-W, *P. aeruginosa*-DSB-S, *P. aeruginosa*-DSB-W, *P. aeruginosa-*OKO-S, *P. aeruginosa*-OKO-W, *P. aeruginosa*-MID-W, *Alcaligenes faecalis*-ILA-S, *P. aeruginosa*-OGU-S, *P. aeruginosa*-UNI-S, *A. faecalis*-MID-S and *P. aeruginosa*. The isolates with a diameter >50 mm include *P. aeruginosa*-MID-W, *P. aeruginosa*-OKO-S and *Pseudomonas* sp.-OWO-S.

**Table 6.** Initial biosurfactant production of the pure isolates

Isolate	OST	Drop collapse test	Emulsification index (E_24_) test (%)
*A. faecalis* (ILA-S) *Pseudomonas* sp*.* (ILA-W)	− +++	− ++	− + (24%)
*P. aeruginosa* (OGU-S) *Pseudomonas* sp. (OGU-W)	− ++	− ++	− + (17%)
*P. aeruginosa* (IDDO-S) *P. aeruginosa* (IDDO-W)	++ +++	++ ++	+ (14%) + (24%)
*P. aeruginosa* (OKO-S) *P. aeruginosa* (OKO-W)	++++ ++	+ +	+ (34%) + (14%)
*P. aeruginosa* (DSB-S) *P. aeruginosa* (DSB-W)	+ ++	+ +	+ (8%) + (28%)
*Escherichia* sp. (MAK-S) *P. aeruginosa* (MAK-W)	+ ++	++ ++	+ (28%) + (16%)
*Pseudomonas* sp. (OWO-S) *Micrococcus* sp. (OWO-W)	++++ ++	++ ++	+ (28%) + (18%)
*P. aeruginosa* (UNI-S) *P. aeruginosa* (UNI-W)	− +	− ++	− + (6%)
*A. faecalis* (MID-S) *P. aeruginosa* (MID-W)	− ++++	− +	− + (28%)
*P. aeruginosa* (AGB-S)	−	−	−
*P. aeruginosa* (AGB-W)	+	++	+ (8%)
Water+crude oil	−	−	−
Detergent+crude oil	++++	+++	+ (57%)
Detergent+olive oil	++++	+++	+ (57%)

**Keys: **Clear zone diameter <5 mm, +; 5 mm, ++; ˂5 mm, -ve; 5 mm, +; ˃5 mm, +++; > 5 mm, ++; <50 mm, ++++; <50 mm, +++.

The results of the emulsification index E_24_ test in Table 6 showed that the highest emulsification index percentage of 34% was recorded in *P. aeruginosa*-OKO-S, followed by *P. aeruginosa*-DSB-W, *Escherichia* sp.*-*MAK-S, *Pseudomonas* sp.-OWO-S and *P. aeruginosa*-MID-W recording 28% and followed by *Pseudomonas* sp*.*-ILA-W and *P. aeruginosa*-IDDO-W recording 24%; *Micrococcus* sp*.*-OWO recorded 18%, *Pseudomonas* sp.-OGU-S recorded 17%, *P. aeruginosa*-MAK-W recorded 16% and *P. aeruginosa-*IDDO-S recorded 14%, while *P. aeruginosa*-UNI-W recorded the least emulsification index percentage of 6%.

### Crude oil degradation using OD for growth measurement

The result in Table 7 showed that *Escherichia* sp.*-*MAK-S recorded the highest increase in growth from 0.436±0.01 nm at day 0 to 1.793±0.01 nm at day 7, and the least growth was recorded by *P. aeruginosa-*IDDO-S which recorded 0.651±0.01 nm at day 0 and 0.834±0.07 nm at day 7; the control recorded little or no growth from 0.333±0.01 nm at day 0 to 0.355±0.01 nm at day 7.

**Table 7.** Crude oil degradation using OD for growth measurement

Isolate	Day 0	Day 3	Day 5	Day 7
*Pseudomonas* sp. (ILA-W)	0.535±0.01	0.618±0.01	0.978±0.05	1.074±0.39
*Pseudomonas* sp. (OGU-W)	0.555±0.01	0.687±0.00	0.899±0.13	0.807±0.01
*P. aeruginosa* (IDDO-S)	0.651±0.01	0.763±0.01	0.980±0.00	0.834±0.07
*P. aeruginosa* (IDDO-W)	0.685±0.01	0.998±0.01	1.104±0.02	1.038±0.01
*P. aeruginosa* (OKO-S)	0.688±0.01	1.376±0.03	1.755±0.04	1.269±0.07
*P. aeruginosa* (OKO-W)	0.331±0.01	0.496±0.00	0.686±0.00	0.769±0.02
*P. aeruginosa* (DSB-S)	0.661±0.02	0.793±0.00	1.255±0.02	0.905±0.02
*P. aeruginosa* (DSB-W)	0.68±0.01	1.052±0.01	2.255±0.01	1.713±0.05
*P. aeruginosa* (MAK-W)	0.432±0.01	0.575±0.00	0.608±0.00	0.658±0.01
*Escherichia* sp. (MAK-S)	0.436±0.01	0.769±0.01	1.739±0.01	1.793±0.01
*Pseudomonas* sp. (OWO-S)	0.432±0.01	0.624±0.00	0.964±0.02	0.984±0.02
*Micrococcus* sp. (OWO-W)	0.535±0.01	0.619±0.00	0.964±0.02	0.935±0.01
*P. aeruginosa* (UNI-W)	0.358±0.01	0.703±0.01	0.749±0.02	0.790±0.21
*P. aeruginosa* (MID-W)	0.688±0.01	1.099±0.02	1.325±0.05	1.168±0.10
*P. aeruginosa* (AGB-W)	0.685±0.01	0.903±0.01	1.244±0.01	0.962±0.02
Control	0.333±0.01	0.394±0.01	0.361±0.01	0.355±0.01

### Changes in OD during crude oil degradation among the high-yield isolates

**Fig. 2.**
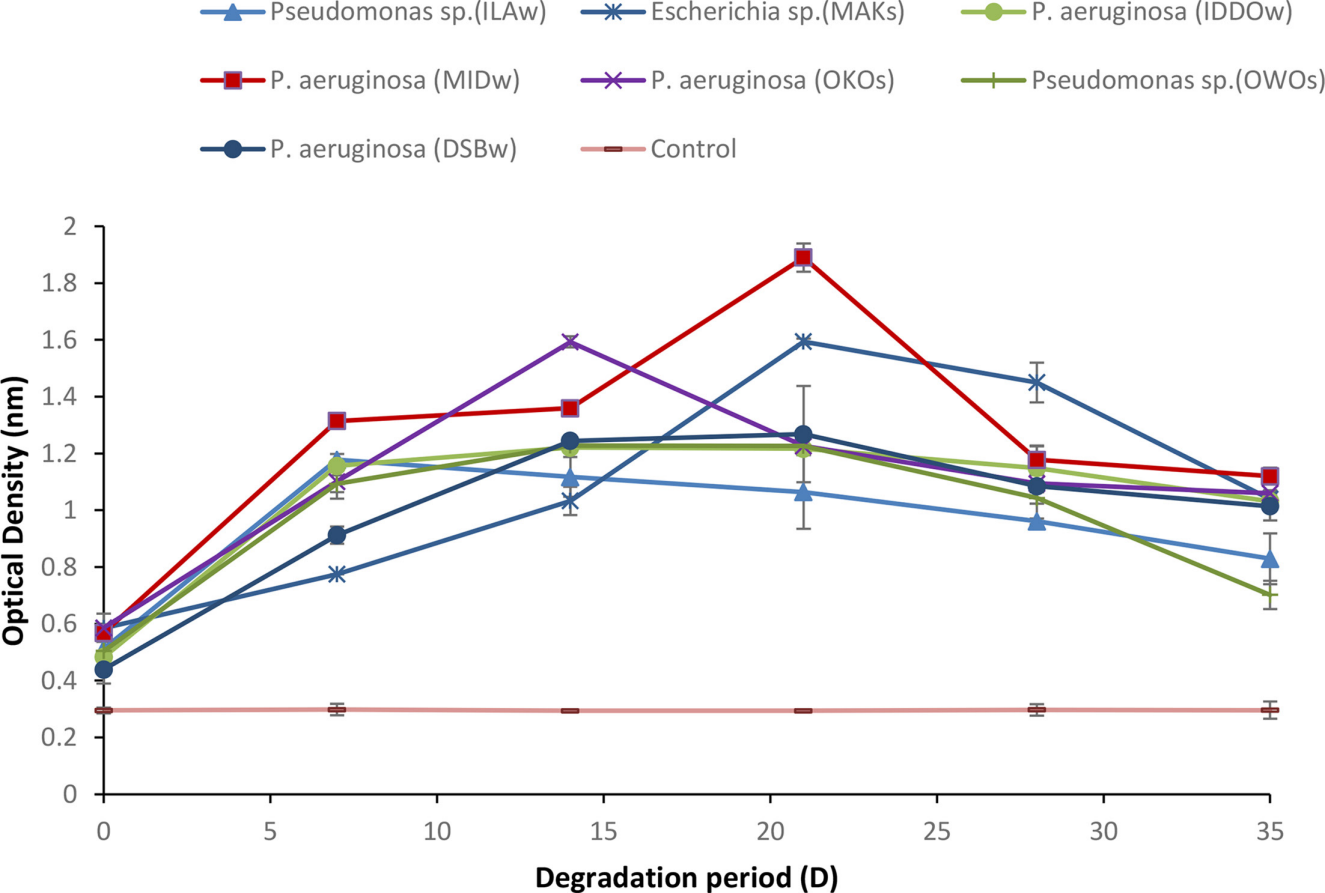
Changes in OD during the degradation of crude oil by the high yield isolates.

The OD results in Fig. 2 revealed that the highest growth rate of 0.568±0.03 nm at day 0 and 1.120±0.03 nm at day 35 was recorded in *P. aeruginosa*-MID-W, followed by *P. aeruginosa*-OKO-S which recorded 0.596±0.05 nm at day 0 and 1.060±0.01 nm at day 35; this was followed by *Escherichia* sp.-MAK-S which recorded 0.585±0.00 nm at day 0 and 1.040±0.02 nm at day 35; the least growth rate among the seven strains was recorded in *Pseudomonas* sp.*-*OWO-S which recorded 0.504±0.00 nm at day 0 and 0.702±0.05 nm at day 35, while there was little or no growth in the control sample.

### Changes in pH during crude oil degradation by the high-yield isolates

**Fig. 3.**
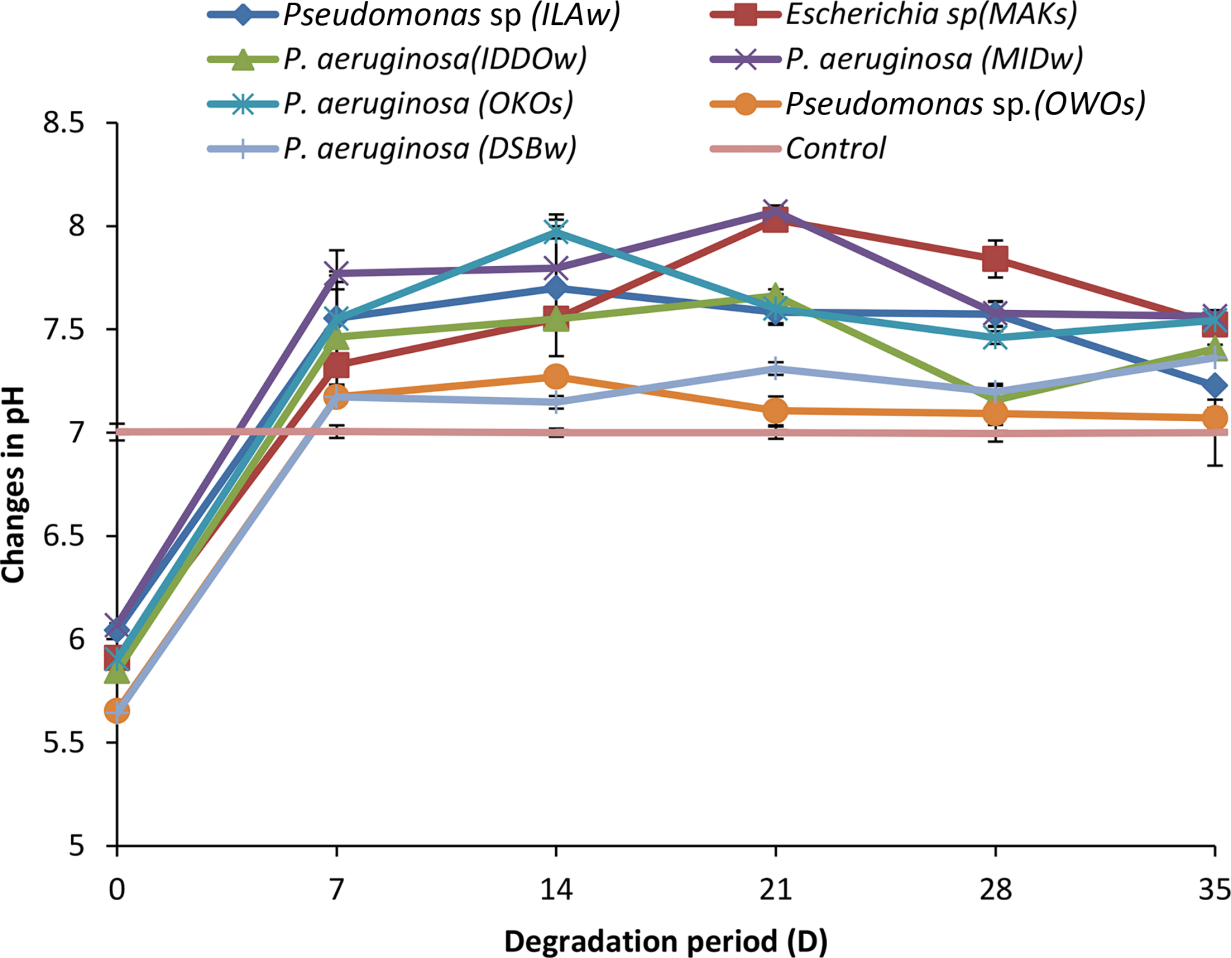
Changes in pH during the degradation of crude oil by the isolates.

The results of the pH in Fig. 3 revealed that there was an increase in the pH values in all the cultures; the highest increase was recorded in *P. aeruginosa*-MID-W having a pH of 6.067±0.01 at day 0 and 7.563±0.03 at day 35; this was followed by *P. aeruginosa* -OKO-S having a pH of 5.903±0.01 at day 0 and 7.543±0.01 at day 35, followed by *Escherichia sp*.*-*MAK-S having a pH of 5.901±0.00 at day 0 and 7.523±0.05 at day 35. The least pH increase among the strains was recorded in *Pseudomonas* sp.-OWO-S having a pH of 6.013±0.03 at day 0 and 7.008±0.05 at day 35, while the control had little or no increase having a pH of 7.005±0.04 at day 0 and 7.005±0.16 at day 35.

### Changes in TPH concentration during crude oil degradation by the high-yield isolates

**Fig. 4.**
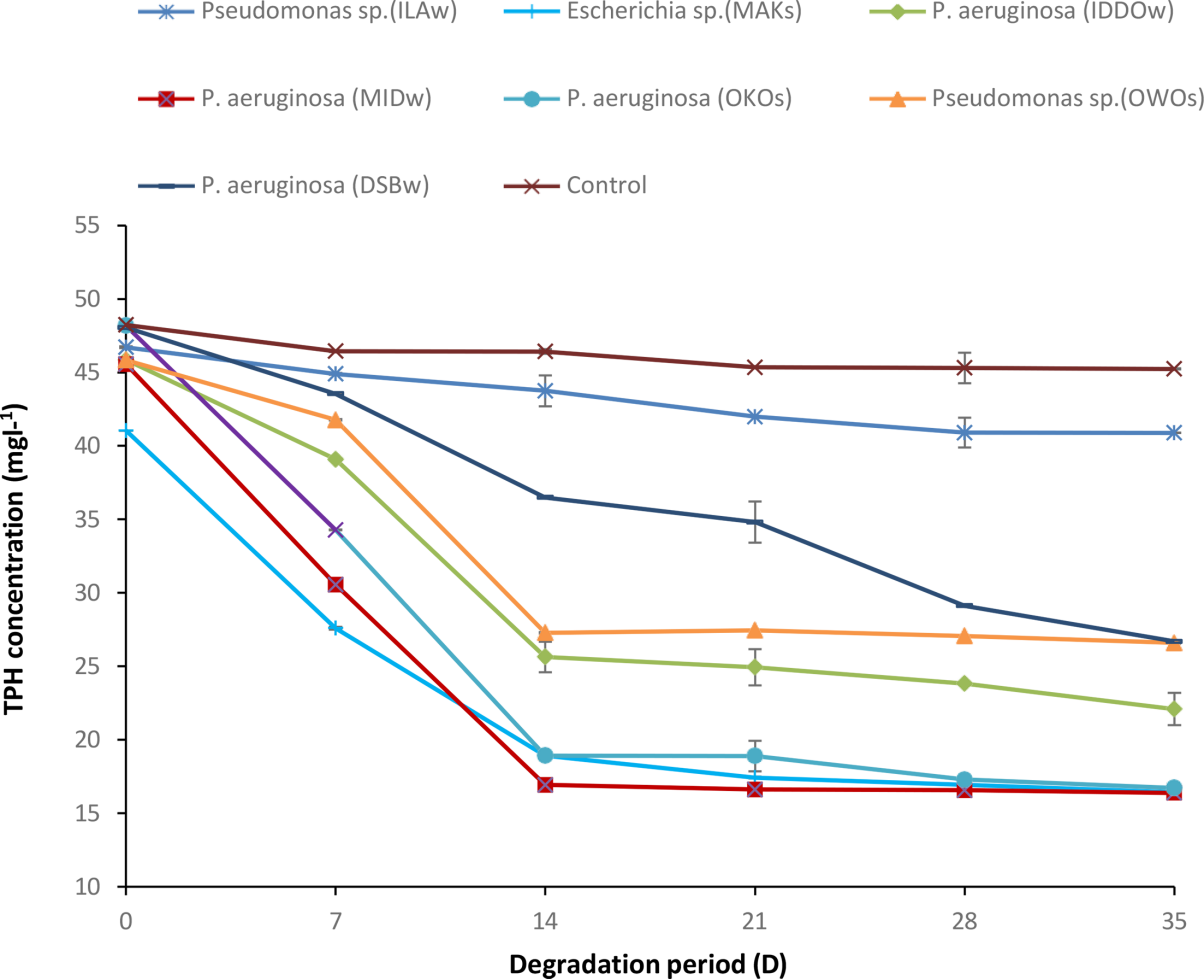
Changes in TPH concentration during the degradation of crude oil by the isolates.

Fig. 4 shows the results of the TPH content analysis. The highest reduction from 48.181±0.07 mg l^−1^ at day 0 to 16.714±0.18 mg l^−1^ at day 35 was recorded in *P. aeruginosa*-OKO-S, followed by *P. aeruginosa-*MID-W which recorded a decrease from 45.652±0.26 mg l^−1^ at day 0 to 16.386±0.01 mg l^−1^ at day 35 and followed by *Escherichia* sp.-MAK-S which recorded a decrease from 41.043±0.01 mg l^−1^ at day 0 to 16.427±0.00 mg l^−1^ at day 35. The least reduction from 46.714±0.06 mg l^−1^ at day 0 to 40.894±0.01 mg l^−1^ at day 35 was recorded in *Pseudomonas* sp.-ILA-W; the control recorded little or no decrease from 48.234±0.03 mg l^−1^ at day 0 to 45.243±0.04 mg l^−1^ at day 35.

### Biosurfactant production using OST

Table 8 shows the biosurfactant production among the seven isolates using OST, and the diameter of cleared zone was recorded in mm. The highest diameter of cleared zone of 40 mm was recorded in *P. aeruginosa*-MID-W and *P. aeruginosa*-OKO-S at day 14, followed by *Escherichia* sp.-MAK-S which recorded a clear zone of 30 mm at day 14. At day 35, there was a decrease in clear zone diameter in *Escherichia* sp.-MAK-S and *P. aeruginosa*-OKO-S to 20 mm, and the clear zone diameter in *P. aeruginosa*-MID-W reduced to 15 mm at day 35. Others including the control tested negative at the end of the experiment.

**Table 8.** Biosurfactant production by the pure isolates using OST analysed as diameter of clear zone (mm)

Organism	Degradation duration (day)					
	0	7	14	21	28	35
*Pseudomonas* sp. (ILA-W)	-ve	-ve	5	10	5	-ve
*Escherichia* sp. (MAK-S)	-ve	15	30	25	25	15
*P. aeruginosa* (IDDO-W)	-ve	5	15	5	5	-ve
*P. aeruginosa* (MID-W)	-ve	20	40	30	25	20
*P. aeruginosa* (OKO-S)	-ve	15	30	30	25	15
*Pseudomonas* sp. (OWO-S)	-ve	-ve	5	5	-ve	-ve
*P. aeruginosa* (DSB-W)	-ve	5	15	20	15	5
Control	-ve	-ve	-ve	-ve	-ve	-ve

### Biosurfactant production potentials using emulsification index E_24_test

Table 9 shows the results of biosurfactant production using emulsification index E_24_ test among the seven isolates; the emulsification index is recorded in percentage (%). The emulsification index of 68% was recorded in *P. aeruginosa*-MID-W at day 14, followed by *Escherichia* sp.-MAK-S and *P. aeruginosa*-OKO-S which both recorded 56% at day 14, but at day 35, there was a decrease in emulsification index percentage in all the strains, while the control tested negative throughout the experiment.

**Table 9.** Biosurfactant production by the pure isolates using emulsification index (E_24_) % test

Emulsification index (E_24_) %						
Organisms	Day 0	Day 7	Day 14	Day 21	Day 28	Day 35
*Pseudomonas* sp. (ILA-W)	10	14	28	20	10	8
*Escherichia* sp. (MAK-S)	17	28	56	24	14	12
*P. aeruginosa* (IDDO-W)	10	13	26	20	10	10
*P. aeruginosa* (MID-W)	17	28	68	24	14	12
*P. aeruginosa* (OKO-S)	17	34	56	30	20	18
*Pseudomonas* sp. (OWO-S)	9	16	32	18	12	10
*P. aeruginosa* (DSB-W)	11	18	36	18	14	10
Control	-ve	-ve	-ve	-ve	-ve	-ve

### Molecular characterization of the bacterial isolates

**Fig. 5.**
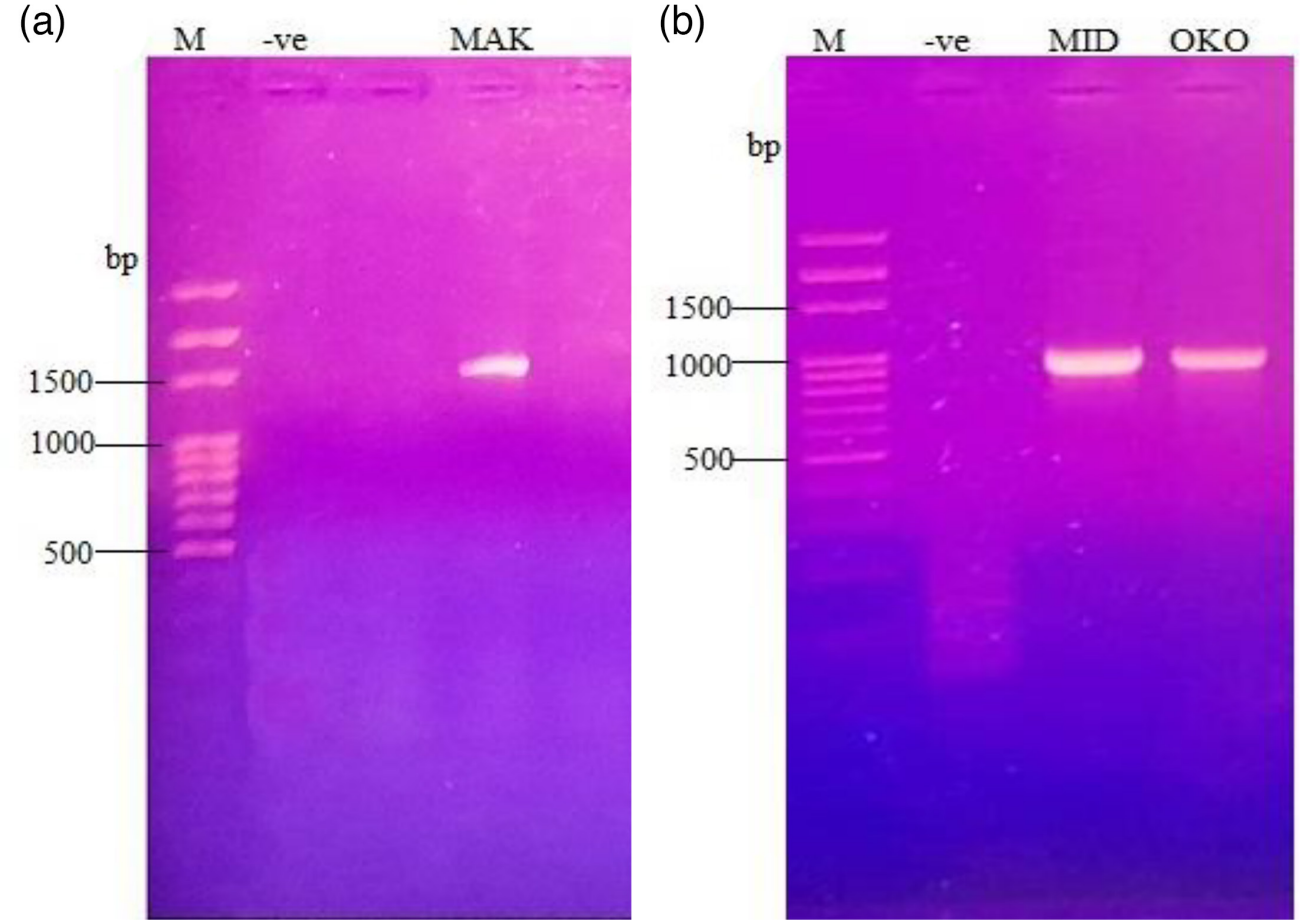
Agarose gel electrophoresis of the PCR product of selected isolates with high potentials for biosurfactant production and crude oil degradation. Lane MAK – *Escherichia* sp. (MAK-S) isolate from Makoko sediment sample (**a**), while lane MID – *P. aeruginosa* (MID-W) and lane OKO – *P. aeruginosa* (OKO-S) isolate from Midlagoon water and Okobaba sediment (b), respectively.

PCR amplification using 16S rRNA universal primer in Fig. 5 reveals band size ~1,500 bp, confirming positive amplification for *Escherichia* sp. (MAK-S) isolate from Makoko sediment sample (Fig. 5a), while Fig. 5(b) reveals band size ~1,000 bp, confirming positive amplification using PASS primer for *P. aeruginosa* (MID-W) and *P. aeruginosa* (OKO-S) isolates from Midlagoon water sample and Okobaba sediment, respectively.

Sequence alignment of the 16S rRNA of MAK-S-WT strain and database search revealed 99% similarity to *Escherichia hermannii* strain K167 by blast analysis; for MID-W-WT strain, the database search revealed 97% similarity to *P. aeruginosa* strain Sihong_820_11, while for OKO-S-WT strain, the database search revealed 99% similarity to *P. aeruginosa* strain P73; these are recorded in Table 10.

**Table 10.** Molecular identification of the isolates

Sample	Location of sample	Nearest strain (through blast)	Accession code of nearest strain	Accession code of strains after submission in GenBank	Percentage (%)
MAK-S	Makoko (sediment)	*E. hermannii* strain K167	JN_644551.1	PQ857166	99
MID-W	Midlagoon (water)	*P. aeruginosa* strain Sihong_820_11	MN_314777.1	PQ857167	97
OKO-S	Okobaba (sediment)	*P. aeruginosa* strain P73	ON_014782.1	PQ857168	99

**Fig. 6.**
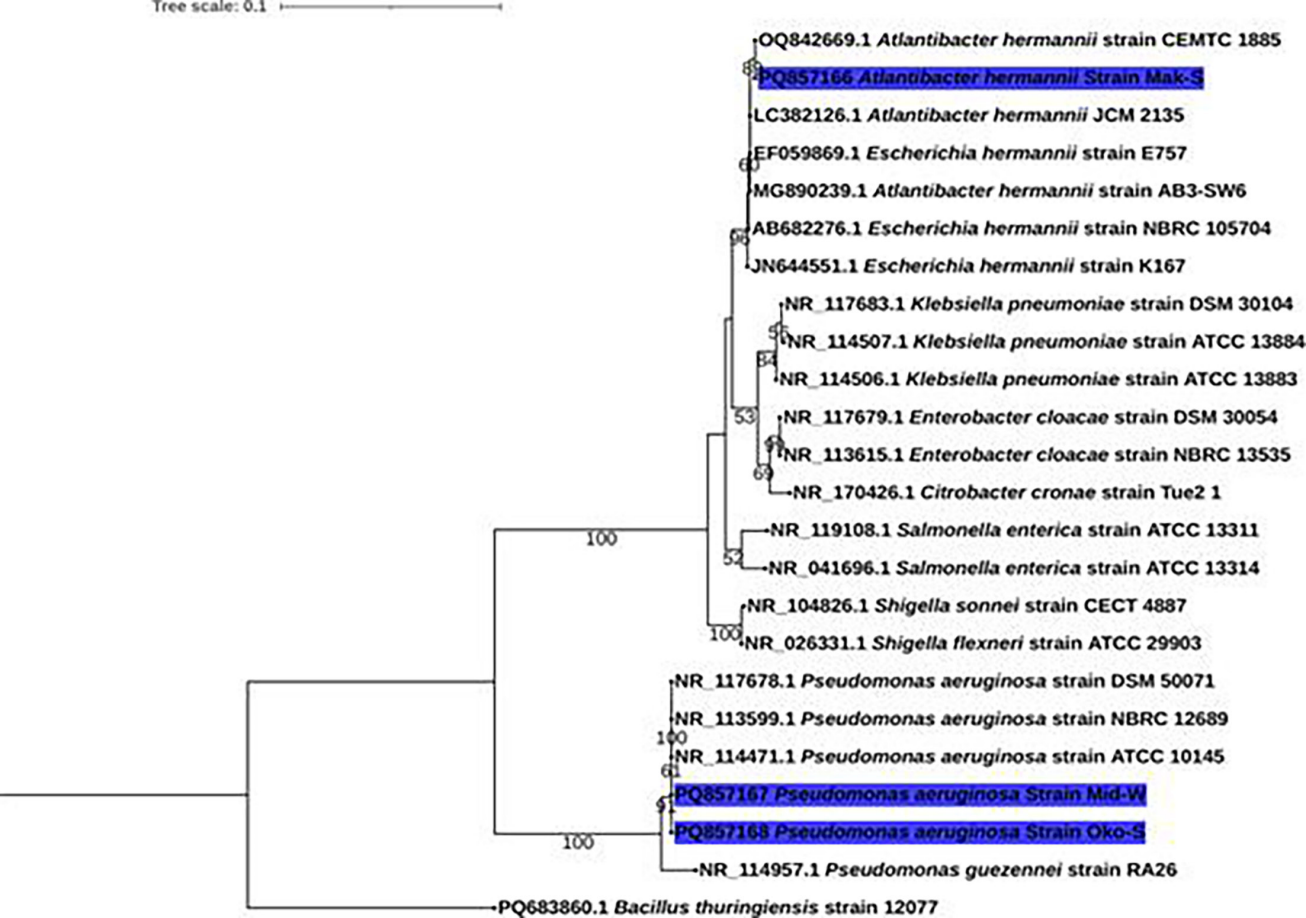
Phylogenetic tree of the bacterial isolates based on the 16S rRNA sequence results.

Based on 16S rRNA sequencing, the phylogenetic tree (Fig. 6) highlights the relationship between the strains that are characterized in this study. There are two clades originating from a common ancestor, one clade with *Bacillus thuringiensis* strains. The other clade consists of two phyletic groups with *P. aeruginosa* strains having a distinct group and with *Pseudomonas guezennei* as an outer group. This group shows that the two *P. aeruginosa* strains, MID-W (PQ857167) and OKO-S (PQ857168), from under-construction sites belong to this group. They share similarities with *P. aeruginosa* strains (DSM 50071, NBRC 12689 and 10145). The other phyletic group consists of strains of *Shigella* sp., *Salmonella* sp., *Citrobacte*r *sp*., *Klebsiella* sp., *Enterobacter* sp., *Escherichia* sp. and *Atlantibacter* sp. *Atlantibacter hermannii* strain Mak-S belongs to this group sharing similarities with *E. hermannii*.

## Discussion

This study investigated biosurfactant-producing bacteria isolated from marine ecosystems for the degradation of crude oil. The physicochemical analysis of the samples revealed that most values for the various parameters were within the permissible limits of the World Health Organization (WHO), while some exceeded the WHO limit for sea water and sediment; this is majorly as a result of pollution of Lagos lagoons.

The highest value of total dissolved solids of 2,014.5 mg l^−1^ was recorded in a sample from Midlagoon; this exceeds the WHO acceptable limit (2000 mg l^−1^), and it is a significant indication of water pollution [25]. Also, *P. aeruginosa* strain Sihong_820_11, one of the organisms with the highest yield in biosurfactant production and crude oil degradation, was isolated from this location. This should be due to the fact that Midlagoon is generally polluted by industrial, domestic and agricultural activities [26]. This lagoon also receives a large quantity of pollutants from manufacturing and municipal activities in the larger Lagos metropolis. Chikwe and Okwa [2] reported that density of human population, ever-increasing numbers of industries and high degree of developmental and agricultural projects around Lagos lagoons have altered the hydrochemical and hydrobiological properties of these water bodies [2,25, 26].

From the morphological and biochemical characterization, the identified isolates, according to [26], belong to the following genera *Pseudomonas, Micrococcus, Alcaligenes* and *Escherichia*. These genera represent a reasonable distribution of microbial diversity, are associated with crude oil and have the ability to degrade numerous hydrocarbons. This agrees with reports [27,28] that marine bacteria isolated and identified to degrade hydrocarbons mostly belong to the species of *Pseudomonas*, *Acinetobacter*, *Alcaligenes*, *Arthrobacter* and *Rhodococcus* [29].

The biosurfactant production analysis using oil spread, drop collapse and emulsification index tests clearly indicates that most of the bacterial isolates can produce biosurfactants, and biosurfactant production efficiency can be up to 34% [30]. These results agree with a former result, stating that for 78 bacteria isolated from petrol pump soil, 52 isolates tested positive for oil displacement test, 43 isolates tested positive for drop collapse test and 36 tested positive for emulsification index test ranging from 10% to 30% [19,30].

From the crude oil degradation by the isolates, using changes in OD, it can be inferred that most of the isolates have the potentials for crude oil degradation, especially *A. hermannii* strain K167 which recorded the highest increase in growth from 0.436±0.01 nm at day 0 to 1.793±0.01 nm at day 7. This also provides a confirmation of the abilities of organisms such as *A. hermannii* strain K167 and *Micrococcus* sp.-OWO-W which recorded 0.535±0.01 and 0.935±0.01 nm at day 7, respectively, to degrade crude oil [27,30]. Seven isolates having high yield for biosurfactant production and crude oil degradation were selected out of the 15 strains for further investigation.

The changes in OD of the seven isolates revealed that all the seven isolates demonstrated microbial growth phases, but the specific periods of the growth phases differ among them, and the increase in the isolates’ growth is directly proportional to the increase in OD [28]. This agrees with a former report stating that the most common way to assess microbial growth in solution is the measurement of the OD at 600 nm [31,32].

There was a progressive increase in the pH values of all the cultures up to the 14th day, after which there was a decrease in pH in all the cultures, but the final pH at day 35 was still higher than the pH values at day 0. This result indicates a strong correlation between the bacterial growth rate using the OD and the pH of the cultures, i.e. the OD of the bacterial cultures is directly proportional to their pH [22]. This is in agreement with a former report that the measurements of OD, pH increase and sulphate reduction efficiency for 21-day incubation revealed that some isolates were capable of growing rapidly with increased OD and pH until after the 21-day incubation period when the estimated exponential phase elapsed [22,33].

The TPH content analysis indicated that most of the isolates effectively utilized the hydrocarbons in crude oil as their sole carbon and energy source, and the hydrocarbon removal efficiencies reached 87% using *P. aeruginosa* strain Sihong_820_11. The variation in rates may be because of factors such as type of organism, environmental conditions, type of hydrocarbon, bioavailability of hydrocarbons and their molecular compositions [34]. This was also reported that the rate of hydrocarbon degradation depends on the creation of enabling environmental conditions to stimulate biodegradative activity, the hydrocarbon type and molecular composition and the bioavailability of hydrocarbons to micro-organisms [35,36].

Based on the comparative study on the biodegradation potentials of the isolates using changes in OD, pH of the cultures and TPH, to a large extent, it can be deduced that *Pseudomonas* spp. have higher capability to degrade hydrocarbons more than other genera [28]. This may be as a result of the fact that *Pseudomonas* spp. are able to grow in simple media and are able to utilize many substrates which may be toxic to other micro-organisms [28]. This is in agreement with a former report that the biochemical versatility of the species of *Pseudomonas* is manifested in the capacity of many of the strains to degrade a number of aliphatic, aromatic, poly-aromatic and other organic compounds [37,38].

Biosurfactant production using OST among the seven isolates showed that the highest diameter was recorded in *P. aeruginosa* strain Sihong_820_11 and *P. aeruginosa* strain P73; this indicates that these two organisms have higher potentials for biosurfactant production among the seven strains [38]. This result also agrees with a former report which states that the oil displacement area in an oil spreading test is directly proportional to the concentration of a given biosurfactant in a solution [39,40].

Among the seven isolates, maximal production of biosurfactants occurred between days 14 and 21 of incubation, the period of the exponential phase of growth for the organisms; also, biosurfactant concentration reduces with a decrease in bacterial growth after day 21. This agrees with the report of [41] that the highest level of biosurfactant production occurred in the exponential phase.

The oil spread and emulsification index (E_24_) tests used to determine biosurfactant production potentials of the isolates revealed that *P. aeruginosa* strain Sihong_820_11 with production efficiency of 68% was observed to have the highest potential for biosurfactant production among the seven strains. *P. aeruginosa* strain P73 and *A. hermannii* strain K167, confirmed by [40,42].

Two strains among the three efficient biosurfactant producers and crude oil degraders were identified molecularly as *P. aeruginosa* strain Sihong_820_11 and *P. aeruginosa* strain P73. This is confirmed in a previous report that *Pseudomonas* species are the major group of organisms colonizing the hydrocarbon-contaminated sites in the environment [41,43]. *A. hermannii* strain K167 is isolated as a biosurfactant-producing and crude oil-degrading bacterium from a marine ecosystem, indicating the possibility of horizontal gene transfer and retention or presence of gene orthologues in this bacterial strain confirming the previous reports of [44].

## Conclusion and recommendation

The results of the present investigation have revealed that biosurfactant-producing bacteria isolated from marine ecosystems in Nigeria can efficiently degrade crude oil. Biosurfactant production efficiency reached 68% using *P. aeruginosa* strain Sihong_820_11 within 2 weeks of incubation. In addition, hydrocarbon removal efficiency was 87% using *P. aeruginosa* strain Sihong_820_11 within 35 days of incubation.

Therefore, it is suggested that efficient biosurfactant-producing and crude oil-degrading bacterial strains were isolated from marine ecosystems in Nigeria. Their genes, especially that of *P. aeruginosa* strain MID-W, can be harnessed to formulate products that will boost the biodegradation of petroleum hydrocarbons in hydrocarbon-contaminated environments.

## Abbreviations

BOD: biological oxygen demand
DCPIP: 2,6-dichlorophenolindophenol
MSM: mineral salts medium
NTU: nephelometric turbidity unit
OST: oil spread test
TPH: total petroleum hydrocarbon
WHO: World Health Organization
